# Retraction Note: LncRNA TUG1 promoted KIAA1199 expression via miR-600 to accelerate cell metastasis and epithelial-mesenchymal transition in colorectal cancer

**DOI:** 10.1186/s13046-021-01855-4

**Published:** 2021-02-04

**Authors:** Junfeng Sun, Jiyi Hu, Guojun Wang, Zhen Yang, Chunlin Zhao, Xiefu Zhang, Jiaxiang Wang

**Affiliations:** 1 Department of Gastrointestinal Surgery, The First Affiliated Hospital of Zhengzhou University, No.1 Jianshe East, Zhengzhou, 450052 China; 2 Colorectal Cancer Center, Fudan University, Shanghai, China; 3 Pediatric Surgery, The First Affiliated Hospital of Zhengzhou University, Zhengzhou, China

**Retraction to: J Exp Clin Cancer Res 37, 106 (2018)**

**https://doi.org/10.1186/s13046-018-0771-x**

The Editor-in-Chief has retracted this article [1] because after publication it was found that the authors did not obtain ethics approval prior to commencing the study. Additionally, concerns were raised regarding a number of image duplications within Figs. 2d, e, 4g and f.

All authors agree to this retraction.
